# Impact and management of physiological calibration in spectral analysis of blood pressure variability

**DOI:** 10.3389/fphys.2014.00473

**Published:** 2014-12-03

**Authors:** Antti M. Kiviniemi, Heidi Hintsala, Arto J. Hautala, Tiina M. Ikäheimo, Jouni J. Jaakkola, Suvi Tiinanen, Tapio Seppänen, Mikko P. Tulppo

**Affiliations:** ^1^Department of Exercise and Medical Physiology, Verve ResearchOulu, Finland; ^2^Center for Environmental and Respiratory Health Research, University of OuluOulu, Finland; ^3^Medical Research Center, University of OuluOulu, Finland; ^4^Institute of Health Sciences, University of OuluOulu, Finland; ^5^Department of Computer Science and Engineering, University of OuluOulu, Finland; ^6^Department of Applied Sciences, London South Bank UniversityLondon, UK

**Keywords:** signal processing, autonomic nervous system, Physiocal, Nexfin, self-adjustment

## Abstract

Physiological calibration (Physiocal) improves the quality of continuous blood pressure (BP) signal from finger. However, the effects of Physiocal on spectral characteristics of systolic BP (SBP) variability are not well-known. We tested the hypothesis that the use of Physiocal may alter the results on SBP variability when compared with BP recording without Physiocal. Continuous BP was recorded simultaneously from fingers of both arms during 10-min standing by two Nexfin devices, one with (ON) and the other without (OFF) Physiocal (*n* = 19). Missing SBP values in ON signal were linearly interpolated over Physiocal sequences (ONinter). The OFF signal was analyzed without any corrections (OFFreference) and after linear interpolation of corresponding sequences when Physiocal appeared in the ON signal (OFFinter). Mean low frequency power of SBP oscillations (LFSBP, 0.04–0.15 Hz) did not differ between the OFFreference, OFFinter, and ONinter. However, LFSBP deviated more from OFFreference when analyzed from ONinter compared with the analysis from OFFinter [median (interquartile range): 14.7 (4.6–38.6) vs. 0.9 (0.5–1.8) %, *p* < 0.05]. In conclusion, the use of Physiocal had a significant effect on the spectral SBP variability that overwhelms the impact of linear interpolation of short data sequences. Therefore, caution is needed when comparing SBP variability between BP datasets acquired with and without Physiocal.

## Introduction

The analysis of blood pressure (BP) variability in frequency domain provides important information on cardiovascular autonomic function in various physiological and clinical settings. More specifically, low frequency (LF, 0.04–0.15 HZ) oscillation in BP, so-called Mayer waves at ~0.1 Hz, has been of great interest due to its relation to cardiovascular sympathetic modulation, baroreflex and, thus, cardiovascular risk (Malliani et al., 1991; Parati et al., 1995; Julien, 2006). Also, high frequency (HF, 0.15–0.4 Hz) fluctuation in BP has been used to assess cardiovascular modulation integrated with respiration (Eckberg, 2003). Together with time-synchronized recording of R-R intervals (RRi), continuous BP measurement enables the assessment of baroreflex sensitivity (BRS) which has provided significant prognostic information in clinical studies (Pagani et al., 1988; La Rovere et al., 1998).

The power of LF and HF oscillation in BP can be calculated from beat-to-beat time series of BP providing satisfactory sampling frequency for short-term spectral analysis. Often, continuous recording of arterial pressure from finger is applied, which has been validated against invasive methods (Imholz et al., 1998). However, LF oscillations of BP seem to amplify with the finger measurement compared with invasive recording which may be explained by complexity of the changes in peripheral vascular state (Omboni et al., 1993). Finger BP measurements involve volume clamp methodology using physiological calibration (Physiocal) criteria to overcome this issue (Wesseling, 1996; Imholz et al., 1998; Bogert and Van Lieshout, 2005). As a trade-off, BP data over ≥ 2 cardiac cycles are lost when Physiolcal is initiated.

During stable physiological conditions, the absence of Physiocal seems to cause no significant drift in BP signal during short-term recording, which is why Physiocal is typically turned off in order to obtain continuous data for the spectral analysis (Omboni et al., 1993; Zhang et al., 2000). However, during acute changes in peripheral vascular state, e.g., cold exposure, this may not be a case (Wesseling et al., 1985; Kurki et al., 1990), when the use of Physiocal seems to be justified. However, the impact of the use of Physiocal on spectral characteristics of BP oscillations is not clear. Typically, artifacts, ectopic beats and signal losses have been managed by interpolating over erroneous data before spectral analysis of variability in beat-to-beat cardiovascular signals (Keselbrener and Akselrod, 1995; Maestri et al., 1998; Salo et al., 2001; Deegan et al., 2011). However, potential bias caused by this correction method on spectral variables of BP variability is unclear. The use of Physiocal and the correction methods to overcome related signal losses may dampen the BP oscillatory waveform or generate artificial variation in BP and, therefore, have varying effects to spectral estimates. Also, it is not known how the often seen difference in BP levels between finger and reference measurement, despite the return-to-flow reconstruction of brachial BP, is taken into consideration before the analyses.

The purpose of the present study was to examine how the use of Physiocal, interpolation over signal losses and correction of deviation in BP from the reference impact the spectral analysis of BP variability and BRS. For this purpose, we continuously recorded BP signal during 10-min standing simultaneously from both arms, one with and the other without Physiocal, the latter being the reference BP recording. Spectral analyses of BP variability and BRS were conducted after linear interpolation over Physiocal sequences and correction for deviation from reference BP level. We hypothesized that the use of Physiocal may alter the results on spectral characteristics of BP variability when compared with BP recording without Physiocal, and BP level correction to match signals with and without Physiocal may decrease these differences in BP variability outcomes.

## Material and methods

### Subjects and study protocol

The 20 participants were recruited by the advertisements on institutional bulletin boards and word of mouth among staff and students at Center for Environmental and Respiratory Health Research (University of Oulu, Oulu, Finland). No special exclusion criteria were applied, as each subject served as his/her own control while conducting measurement simultaneously from adjacent arms. Finally, 19 subjects (13 men, age: 34 ± 10 years, height: 174 ± 8 cm, weight: 72 ± 13 kg) were included, whereas one participant's data were rejected due to the technical problems in the measurements. The study was performed according to the Declaration of Helsinki, and belonged to a larger experimental study (www.clinicaltrials.gov, ID: NCT02007031), where cardiovascular responses to acute cold exposure were studied in population-based samples of normotensive and hypertensive subjects (Hintsala et al., 2014). The present study was motivated by the acute hemodynamic changes (vasoconstriction) expected in cold environment that may affect the finger BP measurement. The study protocol was approved by the local research ethics committee of the Northern Ostrobothnia Hospital District. All subjects gave their written informed consent for participating to the study.

The participants were invited to the thermal laboratories at Kastelli Research Centre (Oulu, Finland). Standard III-lead ECG (Cardiolife, Nihon Kohden, Japan), and finger arterial pressure from both arms (Nexfin, BMEYE Medical Systems, the Netherlands) were continuously recorded during 10-min standing using a PowerLab data acquisition system (Labchart 7 software and PowerLab/8SP, ADInstruments, Australia) with a sampling frequency of 1000 Hz. Nexfin device uses volume clamp methodology and Physiocal criteria, reconstructs finger BP to brachial BP and adjusts for vertical difference between the finger and heart based on height sensor. Standing position was selected because of the main project where cardiovascular responses to cold exposure were assessed particularly during upright position. The measurements were performed in a climatic chamber which temperature was set to thermoneutral conditions (23°C) and enabling adequate peripheral circulation. After instrumentation at sitting position, the participants stood up, having arms supported at heart level. The concordance with systolic (SBP) and diastolic BP (DBP) measured from different arms was inspected, having Nexfin Physiocal feature on. If difference in SBP or DBP was larger than 5 mmHg, the finger cuffs were adjusted. Once SBP and DBP matched between the arms, the signals were monitored for 2 min. Thereafter, Physiocal was turned off randomly from Nexfin placed on either arm and 10-min recording was started. In case of signal loss due to the reason other than Physiocal, the measurement was restarted.

### Data pre-processing

**Figure 1:**
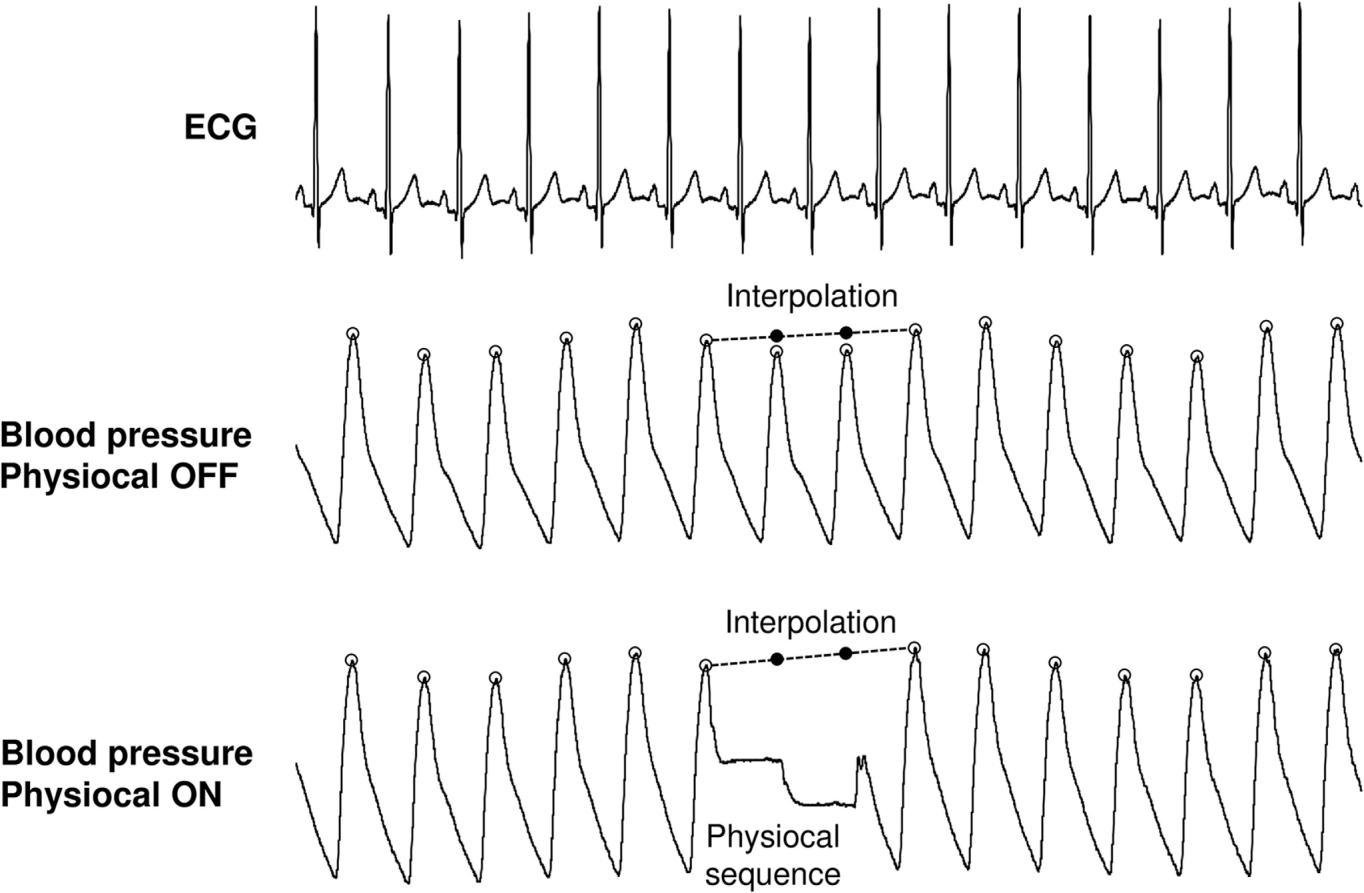
**Processing of continuous blood pressure signals with Physiocal feature off and on**. Physiocal sequence was typically 2–3 beats. The missing systolic blood pressure values were linearly interpolated from last accepted systolic value to the next. This was also conducted to the signal without Physiocal to establish the effects of interpolation.

The BP and ECG signals were exported to custom-made Matlab-based software. The BP signal having Physiocal off (Figure 1, middle panel) was processed, firstly, by performing no corrections (OFF_reference_) and, secondly, by linearly interpolating the sequences, when Physiocal occurred in the BP signal of the other arm (OFF_inter_). Based on previous reports, it was assumed that absence of Physiocal causes no drift in BP signal during short-term recording at stable physiological conditions (Omboni et al., 1993; Zhang et al., 2000) and, thus, OFF_reference_ was defined as reference signal in the present study.

For the BP data obtained from the recording having Physiocal on (Figure 1, lower panel), acceptable SBP values were first identified. Thereafter, the SBP data missing due to Physiocal were linearly interpolated between qualified SBP values around the Physiocal sequences (ON_inter_).

Additionally, the BP data obtained from the recording with Physiocal on were corrected to match SBP level with the OFF_reference_. The raw BP signal having Physiocal on was multiplied by the factor that was calculated by dividing the mean SBP of OFF_reference_ by the mean SBP of signal with Physiocal on, discarding the Physiocal sequences in Labchart 7 software (ADInstruments, Australia). This procedure assumes that the amplitude of SBP oscillation is linearly related to the mean SBP. The BP corrected data with Physiocal sequences were exported from the Labchart 7 software to the custom-made Matlab-based analysis software where linear interpolation between qualified SBP values around the Physiocal sequences was applied (ON-BPcorr_inter_).

### Data analysis

The data were analyzed by custom-made stand-alone Matlab-based program. Time series of RRi and beat-to-beat SBP values were extracted from the continuous ECG and BP recordings that were pre-processed by the methods described above. These discrete event series were then resampled at 2 Hz (Taskforce, 1996). Power spectral analyses of RRi and SBP variability were performed using a fast Fourier transform (Welch method) where segments of 128 samples overlapped in 50% steps throughout the analyzed period. The power spectrum densities of the low (LF, 0.04–0.15 Hz) and high frequency (HF, 0.15–0.4 Hz) oscillations in RRi and SBP were calculated as absolute (ms^2^, mmHg^2^). BRS was analyzed from the LF band by using the alpha method (Pagani et al., 1988). The number of Physiolcal sequences was also calculated.

### Statistical analysis

The data are presented as the median (interquartile range) and mean ± *SD*. In addition, the absolute (|difference|) and relative deviation (|difference|%) from the OFF_reference_ values were calculated. The deviation was chosen because the use of Physiocal and signal pre-processing may either dampen or generate arbitrary SBP oscillatory waveforms. The spectral values of RRi and SBP oscillation were not normally distributed. Therefore, Friedman test was used to assess the main effect of pre-processing methods on the measured variables and their deviations from the reference. Wilcoxon test, corrected for multiple comparisons (*p*-value multiplied the number of comparisons), was used as post-hoc. Furthermore, Spearman correlations were used assess the association of 1) SBP variability from OFF_reference_ to SBP variability from OFF_inter_, ON_inter_ and ON-BPcorr_inter_ signals, and 2) the number of Physiocal sequences and the amount of SBP variability to absolute and relative deviations in LF_SBP_ and HF_SBP_ obtained from OFF_inter_, ON_inter_, and ON-BPcorr_inter_ signals. Finally, Bland-Altman plots were formed to illustrate the agreement between the methods. The data were analyzed using IBM SPSS Statistics 21 (IBM Corporation, Somers, New York). A *p*-value < 0.05 was considered statistically significant.

## Results

Median number of Physiocal sequences was 12 (interquartile range: 12–15). The median and mean values of measured variables are presented in Table 1. On the average, ~4 mmHg higher mean SBP was observed in Physiocal ON signal compared with Physiocal OFF signals. The LF_SBP_ and BRS_LF_ did not differ between the signals or pre-processing methods. Lower HF_SBP_ and greater BRS_HF_ were obtained from OFF_inter_ than OFF_reference_. Also, lower HF_SBP_ and greater BRS_HF_ were observed with ON-BPcorr_inter_ compared with ON_inter_ without BP correction. Noteworthy, HF_SBP_ and BRS_HF_ were closer to OFF_reference_ when ON signal was corrected for the difference in mean SBP.

**Table 1:** **The median values of systolic blood pressure and its variability, heart rate variability and baroreflex sensitivity after different signal pre-processings**.

**Variable**	**OFF_reference_**	**OFF_inter_**	**ON_inter_**	**ON-BPcorr_inter_**	**Main effect**
**SBP, mmHg**					
*Median (IQR)*	123 (114–134)	123 (114–134)	127 (122–133)^*†^	–	0.007
*Mean* ± *SD*	125 ± 12	125 ± 12	129 ± 11	–	
**LF_SBP_, mmHg^2^**					
*Median (IQR)*	9.6 (6.6–17.8)	9.6 (6.6–17.0)	11.7 (7.7–18.5)	10.7 (7.3–18.4)	0.052
*Mean* ± *SD*	18.6 ± 23.3	18.3 ± 22.9	19.1 ± 22.3	17.8 ± 20.5	
**HF_SBP_, mmHg^2^**					
*Median (IQR)*	4.6 (3.4–8.1)	4.7 (3.4–7.5)^*^	4.7 (4.3–10.3)	4.5 (3.8–8.8)^‡^	0.026
*Mean* ± *SD*	5.9 ± 4.4	5.7 ± 4.3	6.4 ± 4.4	6.0 ± 4.1	
**LF_RRi_, ms^2^**					
*Median (IQR)*	446 (279–1236)	–	–	–	–
*Mean* ± *SD*	1105 ± 1677	–	–	–	
**HF_RRi_, ms^2^**					
*Median (IQR)*	235 (44–387)	–	–	–	–
*Mean* ± *SD*	332 ± 419	–	–	–	
**BRS_LF_, ms·mmHg^−1^**					
*Median (IQR)*	7.8 (3.8–10.5)	7.7 (3.9–10.5)	6.8 (3.2–10.3)	7.5 (3.3–10.5)	0.052
*Mean* ± *SD*	7.1 ± 3.7	7.2 ± 3.7	7.0 ± 3.9	7.2 ± 4.1	
**BRS_HF_, ms·mmHg^−1^**					
*Median (IQR)*	5.5 (3.0–8.8)	5.7 (3.1–8.9)^*^	5.1 (2.8–9.2)	5.2 (2.9–9.5)^‡^	0.022
*Mean* ± *SD*	6.9 ± 5.0	7.0 ± 5.1	6.4 ± 4.4	6.6 ± 4.6	

*Values are median (interquartile range, IQR) and mean ± SD. OFF signal with Physiocal OFF, ON signal with Physiocal ON, BPcorr blood pressure level corrected to match with mean of blood pressure signal acquired with Physiocal OFF, inter Physiocal sequences corrected by linear interpolation, SBP, systolic blood pressure; LF, low frequency (0.04–0.15 Hz); HF, high frequency (0.15–0.4 Hz); RRi, R-R interval; BRS, baroreflex sensitivity by alpha method*.

* *p < 0.05 vs. OFF_reference_*,

† *p < 0.05 vs. OFF_inter_*,

‡ *p < 0.05 vs. ON_inter_*.

When inspecting the deviations from the OFF_reference_ in the measured variables, the smallest deviation was constantly observed with OFF_inter_, denoting the significant contribution of the arm of measurement and use of Physiocal in this respect (Table 2). The deviations from OFF_reference_ that were observed with Physiocal ON signals could not be overcome by BP level correction. However, BP level correction significantly decreased the relative deviation from OFF_reference_ in HF_SBP_ and tended so with BRS_HF_ and absolute HF_SBP_.

**Table 2:** **The median values of absolute and relative deviation in systolic blood pressure and its variability, and baroreflex sensitivity using signal without Physiocal as reference**.

**Variable**		**OFF_inter_**	**ON_inter_**	**ON-BPcorr_inter_**	**Main effect**
**LF_SBP_**	**mmHg^2^**	0.11 (0.05–0.20)	1.85 (0.51–6.69)^*^	1.74 (0.39–5.20)^*^	<0.001
	**%**	0.9 (0.5–1.8)	14.7 (4.6–38.6)^*^	11.9 (5.6–31.1)^*^	<0.001
**HF_SBP_**	**mmHg^2^**	0.21 (0.08–0.31)	1.07 (0.68–1.99)^*^	0.71 (0.34–1.84)^*§^	<0.001
	**%**	3.7 (2.1–5.9)	24.6 (16.0–29.2)^*^	17.6 (7.8–22.8)^*†^	<0.001
**BRS_LF_**	**ms·mmHg^−1^**	0.03 (0.01–0.07)	0.26 (0.14–1.19)^*^	0.47 (0.10–1.12)^*^	<0.001
	**%**	0.5 (0.3–0.9)	8.3 (2.4–17.1)^*^	5.7 (2.9–16.5)^*^	<0.001
**BRS_HF_**	**ms·mmHg^−1^**	0.11 (0.03–0.22)	0.56 (0.32–1.12)^*^	0.49 (0.14–0.74)^*§^	<0.001
	**%**	1.9 (1.1–3.1)	10.6 (7.1–15.2)^*^	7.8 (4.1–13.4)^*§^	<0.001

*Values are median (interquartile range). ON signal with Physiocal ON, BPcorr blood pressure level corrected to match with mean of blood pressure signal acquired with Physiocal OFF, inter Physiocal sequences corrected by linear interpolation, SBP, systolic blood pressure; LF, low frequency (0.04–0.15 Hz); HF, high frequency (0.15–0.4 Hz); BRS, baroreflex sensitivity by alpha method*.

* *p < 0.05 vs. OFF_inter_*,

† *p < 0.05 vs. ON_inter_*,

§ *p < 0.1 (tendency) vs. ON_inter_*.

**Figure 2:**
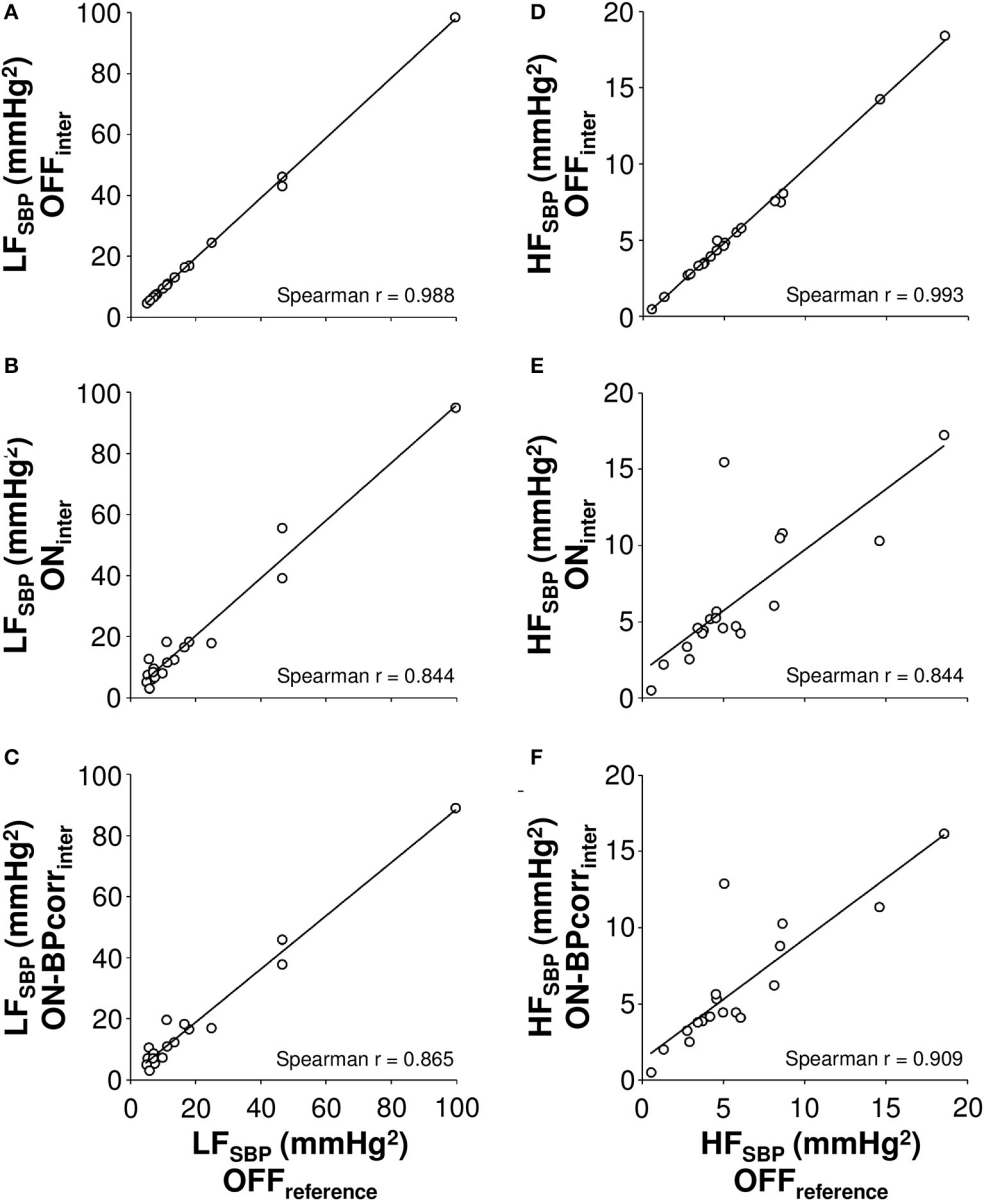
**The correlations of low (LF, 0.04–0.15 Hz, A–C) and high frequency (HF, 0.15–0.4 Hz, D–F) powers of systolic blood pressure (SBP) oscillations, as measured after different pre-processing of Physiocal sequences to the values obtained by the reference measurement**. *OFF* signal with Physiocal OFF, *ON* signal with Physiocal ON, *BPcorr* blood pressure level corrected to match with mean of blood pressure signal acquired with Physiocal OFF, *inter* Physiocal sequences corrected by linear interpolation.

**Figure 3:**
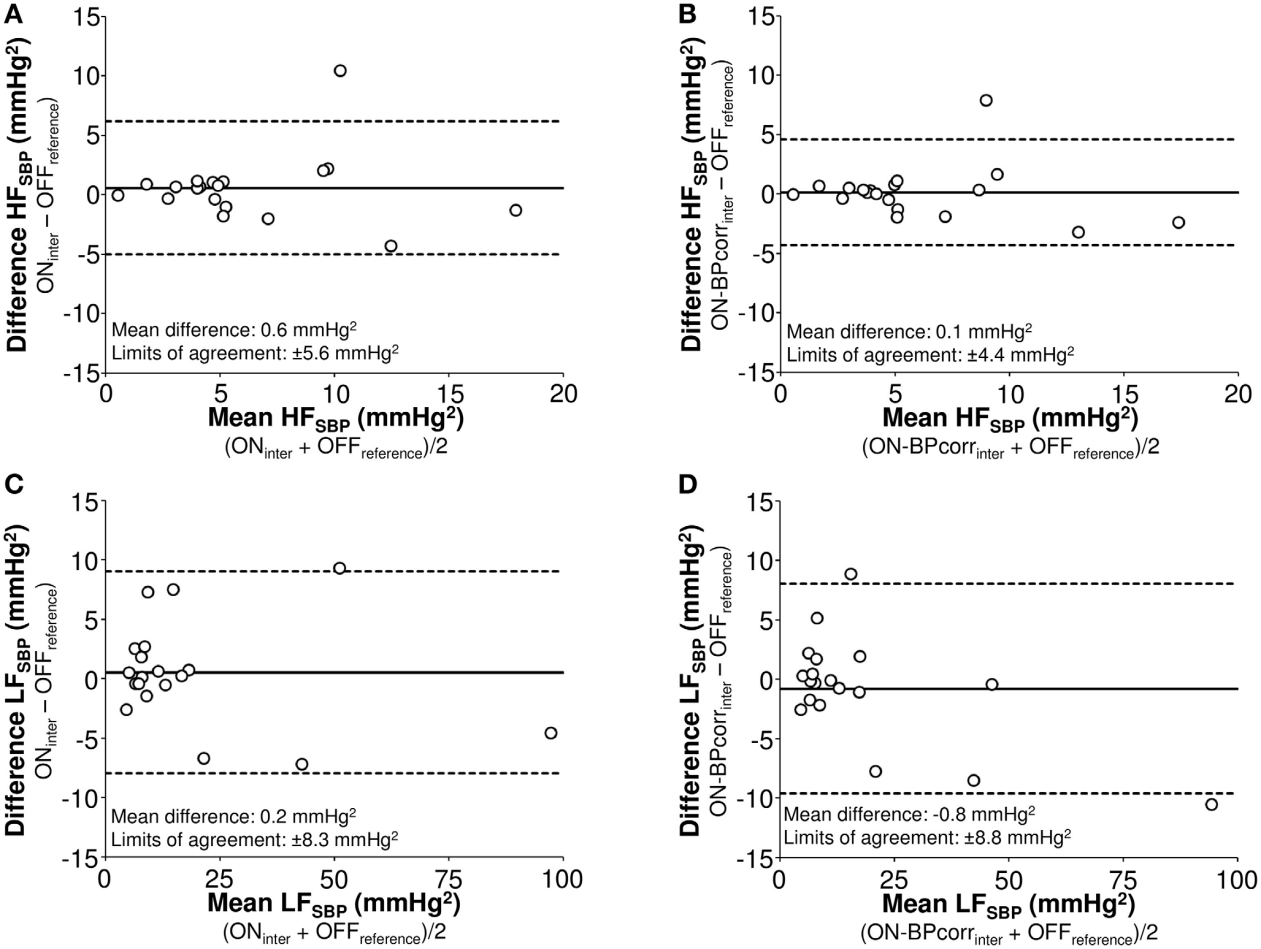
**Bland-Altman plots of high (HF, 0.15–0.4 Hz, A,B) and low frequency (LF, 0.04–0.15 Hz, C,D) power of systolic blood pressure (SBP) variability analyzed from signal with Physiocal on after interpolation (A,C) and after blood pressure level correction and interpolation (B,D) using the values acquired from the recording without Physiocal and interpolation as reference**. *OFF* signal with Physiocal OFF, *ON* signal with Physiocal ON, *BPcorr* blood pressure level corrected to match with mean of blood pressure signal acquired with Physiocal OFF, *inter* Physiocal sequences corrected by linear interpolation.

Spearman correlation analyses showed that BP level correction slightly improved the correlations between Physiocal OFF and ON signal in LF_SBP_ and HF_SBP_ (Figure 2). The BP level correction also decreased the limits of agreement in HF_SBP_ (from ± 5.6 to ± 4.4 mmHg^2^) but not in LF_SBP_ when using OFF_reference_ as reference method (Figure 3).

The deviations of LF_SBP_ and HF_SBP_, obtained by different pre-processing methods, from OFF_reference_ values were not related to the number of Physiocal sequences (*r* = −0.273–0.166). The level of LF_SBP_ from OFF_reference_ explained the absolute deviation in LF_SBP_ caused by interpolation of Physiocal OFF signal (*r* = 0.728, *p* < 0.001) but not the relative deviation or deviations of LF_SBP_ in Physiocal ON signals. It was constantly observed that the greater HF_SBP_ in OFF_reference_ was, the greater was the absolute deviation from OFF_reference_ values with any pre-processing method (*r* = 0.680–0.761, *p* < 0.01). However, such associations were not observed with relative deviations (*r* = 0.104–0.254).

## Discussion

The present study showed that the use of Physiocal has a significant effect on the spectral components of SBP variability and BRS that overwhelms the impact of linear interpolation of short data sequences. The recording with Physiocal resulted in higher mean SBP. However, the BP level correction decreased only slightly the deviation in spectral estimates of SBP variability and BRS caused by the use of Physiocal. Therefore, caution is needed when comparing SBP variability and BRS between BP datasets acquired with and without Physiocal.

The advances in noninvasive continuous BP monitoring have been of pivotal importance in the research on cardiovascular oscillation and autonomic regulation. Currently, manufacturers provide validated reliable equipment for this purpose based on BP measurement from finger. The present study was designed to assess how BP signal loss in for few seconds (2–3 beats), caused by Physiocal, and deviation of BP level from the reference should be handled before spectral analysis which assumes continuous data. Physiocal is intended to improve BP signal quality based on detection of changes in finger vascular state (Wesseling, 1996). Despite Physiocal, re-construction of brachial BP, and other signal processing features of validated noninvasive BP measurement from finger (Eeftinck Schattenkerk et al., 2009), it is commonly observed in practice that BP may differ considerably between finger and brachial measurements, as well as, between the finger measurements from different arms. Whereas finger vascular state may explain this discrepancy, heating of finger (Tanaka and Thulesius, 1993), and re-application of finger cuff are the practical choices of action. In the present study, the use Physiocal may have prevented drift in BP signal to some extent because higher BP was observed when Physiocal was on compared with being off, regardless of stationary physiological conditions, where significant drift is not typically observed when Physiocal is turned off (Omboni et al., 1993; Zhang et al., 2000). This may not be explained by potential physiological or anatomical differences in BP between the arms, as Physiocal was randomly used in either arm.

The principal finding of the present study was that the use of Physiocal during BP recording from a finger has an evident impact on spectral estimates of SBP variability and BRS which was not explained by the interpolation over signal losses caused by Physiocal, and which could not be overcome by correction for observed difference in SBP level. Therefore, BP variability and BRS values cannot be compared interchangeably between data collected with and without Physiocal. The observed deviation in SBP variability and BRS seems to be more complex than simple difference in mean BP that would linearly relate to amplitude of SBP oscillations. In addition to drift in SBP, some decay of unknown origin, altering specifically the amplitude of SBP oscillations, may have occurred in the BP signal without Physiocal. Therefore, BP signal with Physiocal off may not have served as optimal and standard reference signal for spectral analyses, despite the promising previous observations (Omboni et al., 1993; Zhang et al., 2000). In contrast to our expectations, the deviation from reference was not related to the number of Physiocal sequences, which may be explained by small variation in the number of Physiocal sequences between recordings. Therefore, it is difficult to estimate how frequently Physiocal may occur in eligible data.

There has been limited data on how interpolations over signal losses due to the Physiocal affect the outcomes in spectral analysis of SBP variability and BRS. Keselbrener and Akselrod have introduced an interpolation method that was applied in two recordings with these signal losses (Keselbrener and Akselrod, 1995). However, they did not validate this against other reference BP signal. Deegan et al. reported how interpolations of signal losses in BP signal affects transfer function estimates of cerebral autoregulation but did not show results on spectral components of BP variability (Deegan et al., 2011). Also, artificial Physiocal sequences were included in the data that were collected without the Physiocal feature. Therefore, the present study provides novel results in this respect. We observed that inclusion of short linearly interpolated sequences in the continuous SBP time series affected significantly less the outcomes in SBP variability and BRS than the use of Physiocal during the recording. The linear interpolation slightly decreased HF_SBP_ but did not alter the LF_SBP_ that is considered more relevant component in research on baroreflex physiology (Taylor and Eckberg, 1996). The interpolation over 2–3 beats may mildly dampen the amplitude of HF_SBP_ oscillations having maximal wavelength of ~7 s; whereas LF_SBP_, typically having wavelength of ~10 s), are not affected. Taken together, as suggested by previous reports on heart rate variability and cerebral autoregulation, linear interpolation seems to safe be method to manage technical artifacts and ectopic beats before spectral analysis of beat-to-beat cardiovascular data (Taskforce, 1996; Salo et al., 2001; Deegan et al., 2011), whereas the use of Physiocal has greater impact on spectral characteristics of SBP variability and BRS.

### Limitations

The present study is limited by the lack of an absolute reference for brachial pressure. Intra-arterial brachial pressure would have provided considerable input to the present design. However, we believe that the present design is unique using the same system to measure continuous BP from both arms and highlights the effects of Physiocal and its management on SBP variability. Instead of linear interpolation, spline and polynomial functions could have been used for the management of signal losses by Physiocal. Linear interpolation is simple and easily employed in practice, and had minimal effect on outcomes in SBP variability. It remains to be established if more sophisticated interpolation methods improve the reliability of the data.

### Conclusions

Physiocal has a significant effect on the spectral components of SBP variability and BRS that overwhelms the impact of linear interpolation of short data sequences. The recording with Physiocal resulted in higher mean SBP but the BP level correction only slightly decreased the deviations in SBP variability and BRS caused by the use of Physiocal. Therefore, caution is needed when comparing SBP variability and BRS between BP datasets acquired with and without Physiocal.

## Author contributions

Antti M. Kiviniemi: Design of the work, analysis and interpretation of data, drafting the work, final approval of work and its integrity. Heidi Hintsala: Design of the work, acquisition, analysis and interpretation of data, drafting the work, final approval of work and its integrity. Arto J. Hautala: Design of the work, interpretation of data, revision and final approval of work and its integrity. Tiina Ikäheimo: Design of the work, interpretation of data, revision and final approval of work and its integrity. Jouni Jaakkola: Design of the work, interpretation of data, revision and final approval of work and its integrity. Tapio Seppänen: Design of the work, interpretation of data, revision and final approval of work and its integrity. Suvi Tiinanen: Design of the work, interpretation of data, revision and final approval of work and its integrity. Mikko P. Tulppo: Design of the work, interpretation of data, revision and final approval of work and its integrity.

## Grants

This study was supported by funding from the Finnish Technology Development Centre (Tekes, Helsinki, Finland) and the Finnish Foundation for Cardiovascular Research (Helsinki, Finland).

### Conflict of interest statement

The authors declare that the research was conducted in the absence of any commercial or financial relationships that could be construed as a potential conflict of interest.

## Abbreviations

BP: blood pressure
BPcorr: signal corrected for deviation in blood pressure
BRS: baroreflex sensitivity
DBP: diastolic blood pressure
HF: high frequency (0.15–0.4 Hz)
inter: Physiocal sequences interpolated
LF: low frequency (0.04–0.15 Hz)
OFF: signal with Physiocal off
ON: signal with Physiocal on
RRi: R-R interval
SBP: systolic blood pressure.
